# Association between the *TCF7L2* rs12255372 (G/T) gene polymorphism and type 2 diabetes mellitus in a Cameroonian population: a pilot study

**DOI:** 10.1186/s40169-015-0058-1

**Published:** 2015-04-23

**Authors:** Dieudonne Nanfa, Eugene Sobngwi, Barbara Atogho-Tiedeu, Jean Jacques N Noubiap, Olivier Sontsa Donfack, Edith Pascale Mato Mofo, Magellan Guewo-Fokeng, Aurelie Nguimmo Metsadjio, Elvis Ndonwi Ngwa, Priscille Pokam Fosso, Eric Djahmeni, Rosine Djokam-Dadjeu, Marie-Solange Evehe, Folefac Aminkeng, Wilfred F Mbacham, Jean Claude Mbanya

**Affiliations:** Department of Biochemistry, Faculty of Science, University of Yaoundé I, Yaoundé, Cameroon; Laboratory for Molecular Medicine and Metabolism, Biotechnology Center, University of Yaoundé I, Yaoundé, Cameroon; Department of Internal Medicine and Specialties, Faculty of Medicine and Biomedical Sciences, University of Yaoundé I, Yaoundé, Cameroon; National Obesity Center, Yaoundé Central Hospital, Yaoundé, Cameroon; Department of Medicine, Groote Schuur Hospital and University of Cape Town, Cape Town, South Africa; Medical Diagnostic Center, Yaoundé, Cameroon; The Canadian Pharmacogenomics Network for Drug Safety (CPNDS), Center for Molecular Medicine and Therapeutics, Department of Medical Genetics, University of British Columbia, Vancouver, Canada; Laboratory for Public Health Research Biotechnologies, Biotechnology Center, University of Yaoundé I, Yaoundé, Cameroon

**Keywords:** Type 2 diabetes, Genetic association, Transcription factor 7-like 2 (*TCF7L2*), Sub-Saharan Africa, Cameroon

## Abstract

**Background:**

To study the relationship between the rs12255372 (G/T) polymorphism of the transcription factor 7-like 2 (*TCF7L2*) and type 2 diabetes mellitus (T2DM) in a Cameroonian population.

**Methods:**

This case–control study included 60 T2DM patients and 60 healthy normoglycemic controls, all unrelated and of Cameroonian origin, aged above 40 years (range 40–87). The Restriction Fragment Length Polymorphism - Polymerase Chain Reaction (RFLP-PCR) was used for genotyping.

**Results:**

The T allele frequency was significantly higher in the diabetic group (0.44) than in the control group (0.17). This allele was significantly associated to a greater risk of developing T2DM as compared to the G allele (OR = 3.92, 95% CI 2.04 – 7.67, p < 0.0001). The codominant (additive) model explained best the risk of developing the disease, as the TT genotype was significantly associated to T2DM when compared to the GG genotype (OR = 4.45, 95% CI 1.64 – 12.83, p = 0.0014). By logistic regression adjusted for age, this OR was 4.33 (95% CI: 1.57 – 11.92, p = 0.005).

**Conclusion:**

Our findings suggest that the rs12255372 (G/T) polymorphism of the *TCF7L2* gene is an important risk factor for T2DM in the Cameroonian population.

## Background

Diabetes is a non-communicable disease characterized by chronic hyperglycemia and disturbances in carbohydrates, lipids and proteins metabolism due to defects in insulin secretion and its action, which results in severe acute and chronic complications [1]. Diabetes mellitus is a major public health problem worldwide. Estimates from the International Diabetes Federation (IDF) indicate that there were about 381.8 million adults with diabetes mellitus in the world in 2013. This prevalence is projected to expand by 55% in 2035 to reach 591.9 million of adults affected [2]. Type 2 diabetes mellitus (T2DM) causes important morbidity, disability and early mortality, and is associated with a huge economic burden [3].

T2DM is the most heterogeneous form of diabetes which is due by interactions between genetic and environmental factors. Genome-wide association studies have led to the identification of several susceptibility genes for T2DM [4], including the gene coding for the transcription factor 7-like 2 (TCF7L2). *TCF7L2* is involved in insulin secretion [5,6]. It intervenes in the “wingless” (WNT) signalization in β pancreatic cells, L cells of the intestine and in adipocytes [7]. An association study reported a relationship between a common micro-satellite (DG10S478) in intron 3 of the *TCF7L2* gene and T2DM [8]. Other studies identified 4 other polymorphisms of the *TCF7L2* gene associated to T2DM, amongst which rs7903146 (C/T), rs7901695 (T/C), rs12255372 (G/T) and rs11196205 (G/C) [9,10]. Even though most studies demonstrated a strong association between the *TCF7L2* rs7903146 (C/T) variants with T2DM, some few have also shown a strong association with the rs12255372 (G/T) variants [11-17]. On the contrary, no association between the rs12255372 (G/T) variants and T2DM have been found in Chinese [18], Arabs [19], Pima Indians [20] and South-Africans [21].

The relationship between the *TCF7L2* variants gene and T2DM has never been studied in Central African populations where T2DM is very prevalent, with high morbidity and mortality rates [2]. Hence we decided to set bases with this pilot study by investigating the association between the *TCF7L2* rs12255372 (G/T) polymorphism and T2DM in a Cameroonian population.

## Methods

### Study population

This is a case–control study involving 60 T2DM patients and 60 non-diabetic controls of Cameroonian ethnicity aged over 40 years. T2DM patients, diagnosed according to the IDF criteria [22], were consecutively recruited through the outpatient clinic of the National Obesity Center of the Yaoundé Central Hospital, Yaoundé, Cameroon. Pregnant or breastfeeding women were excluded. Non-diabetic controls were recruited from the general population and included in the study after being tested negative for diabetes (fasting plasma glucose <126 mg/dL) [22].

For all participants, we collected data on the sex and age; height, waist and hip circumference to the nearest 0.5 cm, and weight in light clothes to the nearest 0.1 kg were measured. The body mass index (BMI) as weight in kg/height^2^ in m^2^, and the waist-to-hip ratio were calculated. The resting blood pressures were measured using standardized procedures with an automatic sphygmomanometer Omron HEM-705 CP (Omron Corporation, Tokyo, Japan).

### Biochemical assays and molecular genotyping

Blood samples were collected for biochemical and molecular assays. Fasting plasma glucose (glucose oxidase–peroxidase method), serum triglycerides (glycerol phosphatase oxidase-phenol4-amino antipyrene peroxidase method), serum cholesterol and high-density lipoprotein (HDL)-cholesterol (cholesterol oxidase phenol4-amino antipyrene peroxidase method) were determined by spectrophotometer (UV Mini 1240) using Chronolab kits (Chronolab Systems, Barcelona, Spain). Low-density lipoprotein (LDL)-cholesterol was calculated using the Friedwald’s formula [23].

Genomic DNA was extracted from whole blood on filter paper by the Chelex method [24] and stored at −20°C. The rs12255372 (G/T) polymorphism of the *TCF7L2* was genotyped by Restriction Fragment Length Polymorphism – Polymerase Chain Reaction (RFLP-PCR) using the following primers: Forward 5′-CTG GAA ACT AAG GCG TGA GG-3′, Reverse 5′-GGG TCG ATG TTG TTG AGC TT-3 (SIGMA-ALDRICH, St. Louis, Missouri, United States). A final reaction volume of 20 μL for the Polymerase Chain Reaction (PCR) was constituted, which contained 100 ng of genomic DNA, 0.25 μM of each primer, 1.5 mM of MgCl_2_, 0.2 mM of each deoxynucleotide triphosphate (dNTP), 0.5 U of Go Green *Taq* DNA polymerase (PROMEGA), 1× Go Green Flexi buffer and 11.3 μl of nuclease free water. The PCR was carried out on a BIOMETRA T3 Thermal Cycler under the following conditions: 95°C for 2 minutes, followed by 35 cycles of 95°C for 30 seconds, 54°C for 30 seconds, 72°C for 30 seconds, and a final extension of 72°C for 5 minutes. The amplicons (346 bp) were then digested with *Thermus species* (*Tsp*509I) restriction enzyme at 65°C for 3 hours. The reaction volume was set to 15 μl, containing 7 μl of amplicons, 1× NEB buffer1 (New England Biolabs), 1U of *Tsp*509I, and 6.3 μl of nuclease free water. The digested products were separated by electrophoresis on a 3.5% agarose gel in presence of ethidium bromide (10 mg/mL) and visualized under a UV transilluminator.

### Ethical considerations

The study was approved by the National Ethical Review Board of the Cameroon Ministry of Public Health. Written informed consent was obtained from all the participants. The study was conducted in accordance with the Helsinki Declaration.

### Statistical analysis

Data was analyzed with STATA 11.0 (STATA Corporation, College Station, Texas, USA). Genotype and allele frequencies were compared using the χ^2^ statistics or the fisher’s exact test. Continuous variables were compared using non parametric tests (Mann Whitney or Kruskall Wallis with post hoc multiple comparison by Dunn-Sidak test). The Hardy Weinberg equilibrium was tested using the goodness-of-fit chi-square. Odd ratios were calculated by logistic regression adjusting for age. A *p* value less than 0.05 was considered statistically significant.

## Results

Significant differences between T2DM patients and normoglycemic controls were observed for age (median age – 60 years vs 50 , *p* < 0.0001), waist-to-hip ratio (median value - 0.96 vs 0.87, *p* < 0.0001), fasting plasma glucose (median level - 1.49 vs 0.91, *p* < 0.0001), total cholesterol (median level - 163.00 vs 191.00, *p* < 0.0001) and LDL-cholesterol (median level - 86.00 vs 110.00, *p* < 0.0001) (Table 1).

**Table 1:** **Clinical and biological characteristics of the study population**

**Variables**	**Normoglycemic individuals (n = 60)**	**Type 2 diabetes patients (n = 60)**	***P*** **value**
Age (years)	50 (45 – 54)	60 (53 – 67)	<0.0001
Male/female ratio	20/40	28/32	0.136
Waist-to-hip ratio	0.87 (0.81 – 0.91)	0.96 (0.91 – 0.98)	< 0.0001
Systolic blood pressure (mmHg)	136 (120 – 152)	130 (118 – 152)	0.70
Diastolic blood pressure (mmHg)	83.5 (76 – 93)	78 (71.25 – 87)	0.034
Body mass index (kg/m^2^)	28.39 (26.05 – 32)	27.85 (25.01 – 33.56)	0.59
Fasting plasma glucose (g/L)	0.91 (0.84 – 1.00)	1.49 (1.27 – 2.08)	<0.0001
Total cholesterol (mg/dl)	191 (173 – 210)	163 (149 – 179)	<0.0001
HDL-cholesterol, mg/dl	50 (46 – 54)	48.5 (44 – 52.75)	0.070
LDL-cholesterol, mg/dl	110 (91.25 – 138.5)	86 (76.25 – 100)	<0.0001
Tryglicerides (mg/dl)	141.5 (129.5 – 161.8)	136 (122 – 157.5)	0.124

Data are medians (interquartile range) unless otherwise stated.

HDL-cholesterol: high density lipoprotein cholesterol; LDL-cholesterol: low density lipoprotein cholesterol.

**Figure 1:**
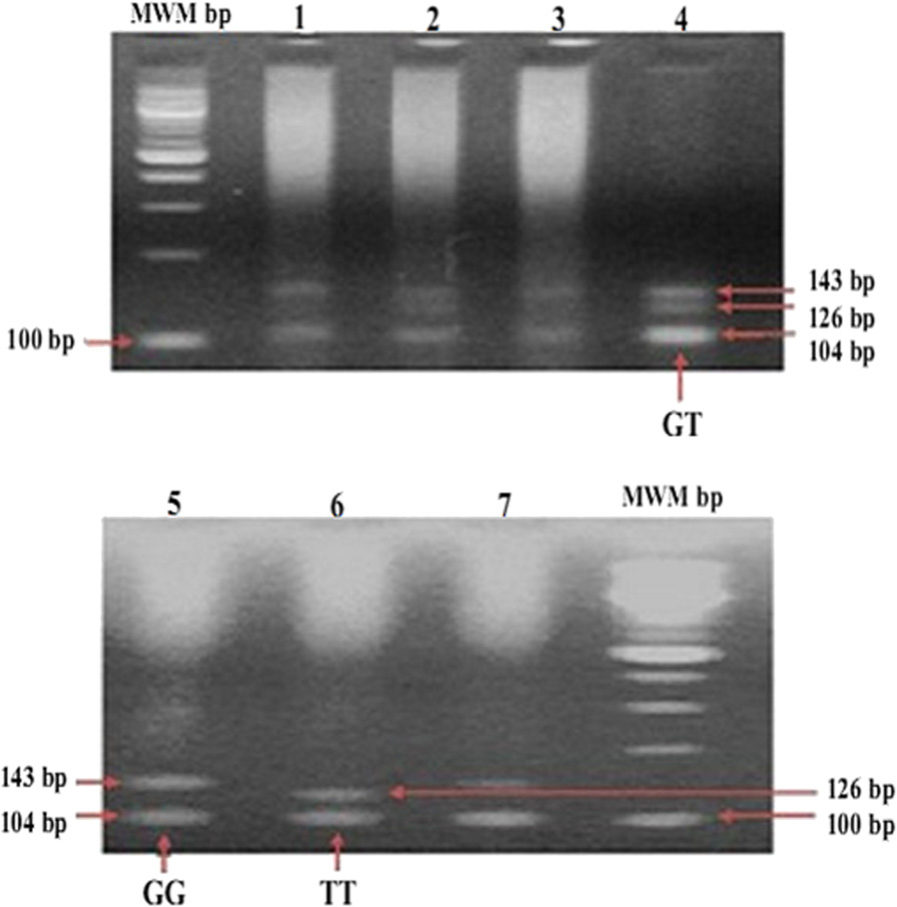
PCR-restriction fragment length polymorphism detection of the *TCF7L2* rs12255372 (G/T) polymorphism. PCR followed by digestion with *TSP*509I - 3.5% agarose gel electrophoresis followed by ethidium bromide staining and UV transilluminator was performed. The expected product sizes are: normal homozygote GG, 143 bp, 104 bp; mutant homozygote TT, 126 bp, 104 bp; and heterozygote GT, 143, 126, and 104 bp, respectively. MWM: 100 bp Molecular weight marker; fragments smaller than 100 bp were not visualized.

From the 120 participants, five cases were excluded from the final analysis because of negative genotypic results. One hundred and fifteen cases were positive for genotyping, characterized on agarose gel by two bands of 143 bp and 104 bp for the wild type homozygote GG, two bands of 126 bp and 104 bp for the mutant homozygote TT, and three bands of 143 bp, 126 bp and 104 bp for the mutant heterozygote GT. Fragments smaller than the 100 bp of the molecular weight marker were not visualized (Figure 1).

The frequency of the GG genotype was 66.96% (77/115), versus 5.21% (6/115) and 27.82% (32/115) for the GT and the TT genotypes respectively. Genotype frequencies violated the Hardy-Weinberg equilibrium in the general population. The G allele was major with a frequency of 70%, as compared to the minor T allele which showed a frequency of 30%. The T allele frequency was 43.96% in diabetic group against 16.7% in nondiabetic and was found to significantly increase the risk of T2DM with an odds ratio of 3.92 (95% CI 2.04 – 7.67, p < 0.0001) (Table 2).

**Table 2:** **Association between the** ***TCF7L2*** **rs12255372 (G/T) polymorphism with type 2 diabetes mellitus**

**rs12255372 (G/T)**	**Controls, n (%)**	**T2DM, n (%)**	**OR (95% CI)**	***p value***
Alleles				
G	95 (83.33)	65 (56.03)	/	/
T	19 (16.7)	51 (43.96)	3.92 (2.04 – 7.67)	0.0001
Total (2 N)	114	116		
Genotypes				
GG	46 (80.7)	31 (53.44)	/	
GT	3 (5.26)	3 (5.17)	1.48 (0.18 – 11.75)	0.68
TT	8 (14.03)	24 (41.37)	4.45 (1.64 – 12.83)/4.33* (1.57 – 11.92)	0.0014/0.005*
Total (N)	57	58		

T2DM: Type 2 diabetes mellitus; OR: odd ratio; *age-adjusted odd ratio.

The frequency of the TT genotype was significantly higher in diabetics than in controls (41.37% vs. 14.03%) and was found to be significantly associated to T2DM with an OR of 4.45 (95% CI 1.64 – 12.83, p = 0.0014) (Table 2).

Dominant, recessive and codominant models of inheritance were tested to identify which of them best fit the effect of the *TCF7L2* rs12255372 (G/T) polymorphism on T2DM. Assuming the dominant model (GG vs. GT + TT = XT), the T allele carriers (XT genotype) had a significantly higher risk of T2DM than those with the GG genotype with an OR of 3.64 (95% CI 1.47 – 9.30, p = 0.0028). When assuming the recessive model (GX = GG + GT vs. TT), a significant association was also found with an OR of 4.32 (95% CI 1.62 – 12.36, p = 0.00016). Thus, the highest risk was observed with the codominant model with an OR of 4.45 (95% CI 1.64 – 12.83, p = 0.0014)/4.33 (95% CI: 1.57 – 11.92, p = 0.005), after adjusting for age (Table 2).

Based on rs12255372 (G/T) genotypes, the clinical (BMI, waist-to-hip ratio, systolic blood pressure and diastolic blood pressure) and biochemical (fasting plasma glucose, total cholesterol, LDL-cholesterol, HDL-cholesterol and triglycerides) characteristics of the T2DM patients were stratified, and no association was found. Comparative analyses of normoglycemic controls and rs12255372 (G/T) genotypes also revealed no association with anthropometrical and biochemical characteristics (data not shown).

## Discussion

Although the burden of T2DM is huge in sub-Saharan populations, epidemiological data on the disease are limited, especially on the genetic determinants of the disease [25]. This pilot study aimed to assess the association between the rs12255372 (G/T) polymorphism of the *TCF7L2* gene with T2DM in a Cameroonian population. The frequency of the minor T allele was found to be 30%, and was comparable to those observed in the Czech population (30.15%) [26], the Iranian population (34.45%) [13] and the Arab population (36.15%) [19]. The variation of the T allele frequency across population could be explained by the genetic diversity between different ethnic groups [14]. This allele was found to be significantly associated to the risk of T2DM with an OR of 3.92 (95% CI 2.04 – 7.67, p < 0.0001). This result is consistent with those reported by previous studies in different populations [13,16,17], where a strong association was noted between this polymorphism and the risk of T2DM. Furthermore, a weak association was reported in West-Africa, with an OR of 1.31 (95% CI 1.01–1.69, P = 0.044) [27] and in Afro-Americans [28]. However, no association between a *TCF7L2* rs12255372 (G/T) variant and T2D was found in Chinese [18], Arab [19], Pima Indians [20], and South-African (Zulu offspring) [21] populations.

The frequency of the TT genotype was significantly higher in diabetic patients than normoglycemic individuals (41.37% vs. 14.03%, p = 0.0014). The GT genotype frequency was similar between the 2 groups and no association was found with of T2D. When assuming 3 models (dominance, recessivity, and codominance) to explain the association between the rs12255372 (G/T) polymorphism of the *TCF7L2* gene and T2D, the codominant model best fitted the association with an OR of 4.45 (95% CI 1.64 – 12.83, p = 0.0014)/OR (adjusted for age) of 4.33 (95% CI: 1.57 – 11.92, p = 0.005), thus a 4-fold risk increase. This finding is in accord with that of Faranak et al. who demonstrated that the codominant model best fitted the effect of these gene variant on the risk of T2DM in the Iranian population [13]. A meta-analysis published in 2009 showed that the magnitude of association between this gene variant and T2DM is moderate and that the TT homozygous variant will approximately cause a 2-fold increase in T2DM [10]. Our value was higher (about 2-times higher), and could have been due to our sample size which was not adequate (small) for this kind of study, as we noted very big confidence intervals for odds Ratio. Despite that, differences in ethnic background, environmental factors such as life-style also could explain the risk difference. Even though the biological mechanism for the association between the *TCF7L2* gene and the risk of T2DM is still unclear, it can be speculated that the *TCF7L2* gene has a role in insulin secretion and possibly adipose tissue development. Furthermore, the genetic variants that have so far been studied are present in the introns rather than in the coding regions. However, this may still lead to functional consequences in terms of protein stability and/or expression of alternatively spliced variants [12].

This study, with a caution on sample size showed an association between the rs12255372 (G/T) polymorphism of the *TCF7L2* gene and type 2 Diabetes in a Cameroonian population. The Hardy-Weinberg equilibrium was violated in the general population, and this could have been due to genotyping errors, as RFLP-PCR data is sometimes difficult to interpret. For this reason, we excluded all doubtful genotypes from analysis. Deviation from Hardy-Weinberg equilibrium in this study was most probably due to our small sample size [29].Thus, our findings have to be replicated with a large sample size and genotyping has to be done using more sensitive techniques such as the Taqman probe assay on real time PCR, or by direct sequencing, as RFLP-PCR data are sometimes difficult to appreciate. Controls have to be carefully selected in other to avoid confounding by population stratification. However, despite the relative small sample size of the study, the high odd ratio and level of statistical significance found are clear indications that there is most probably an association between rs12255372 *TCF7L2* and T2DM in our population.

## Conclusion

The rs12255372 (G/T) polymorphism of the *TCF7L2* gene is probably associated with T2DM in this population. This variant could help to predict the occurrence of T2DM in the Cameroonian population and possibly other sub-Saharan populations. Our findings should be confirmed by larger study with more accurate genotyping tools.

## Abbreviations

*TCF7L2*: Transcription factor 7-like 2
T2DM: Type 2 diabetes mellitus
BMI: Body mass index
OR: Odd ratio
HDL: High-density lipoprotein
LDL: Low-density lipoprotein
